# Do we still need animals? Surveying the role of animal‐free models in Alzheimer’s and Parkinson’s disease research

**DOI:** 10.15252/embj.2021110002

**Published:** 2022-02-24

**Authors:** Liesbeth Aerts, Beatrice Miccoli, Aaron Delahanty, Hilda Witters, Sandra Verstraelen, Bart De Strooper, Dries Braeken, Patrik Verstreken

**Affiliations:** ^1^ VIB Center for Brain & Disease Research VIB Leuven Belgium; ^2^ KU Leuven – University of Leuven Leuven Belgium; ^3^ IMEC vzw Leuven Belgium; ^4^ Flemish Institute for Technological Research, Unit Health VITO Mol Belgium; ^5^ UK Dementia Research Institute at University College London London UK

**Keywords:** 3R, Alzheimer’s, animal research, non‐animal methods, Parkinson’s, replacement, Neuroscience, Science Policy & Publishing

## Abstract

The use of animals in neuroscience and biomedical research remains controversial. Policy is built around the “3R” principle of “*Refining, Reducing and Replacing*” animal experiments, and across the globe, different initiatives stimulate the use of animal‐free methods. Based on an extensive literature screen to map the development and adoption of animal‐free methods in Alzheimer's and Parkinson's disease research, we find that at least two in three examined studies rely on animals or on animal‐derived models. Among the animal‐free studies, the relative contribution of innovative models that may replace animal experiments is limited. We argue that the distinction between animal research and alternative models presents a false dichotomy, as the role and scientific value of both animal and animal‐free approaches are intertwined. Calls to halt all animal experiments appear premature, as insufficient non‐animal‐based alternatives are available and their development lags behind. In light of this, we highlight the need for objective, unprejudiced monitoring, and more robust performance indicators of animal‐free approaches.

## Graphical abstract

The use of animals in biomedical research is centered around the 3R principle of *Refining, Reducing and Replacing* animal experiments, but is the field ready to implement animal‐free research for good?

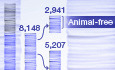

## Introduction

Animal testing in biomedical research is under increased scrutiny worldwide, with more and more societal and political pressure to refine, reduce, and replace the use of animals in experiments. In addition to ethical and animal welfare concerns, critics of animal research claim that animal models offer limited translational value (Pound & Ritskes‐Hoitinga, 2018), and advocate a complete shift to human‐centered methods. Dedicated legislation is in place to maximize animal welfare (Box 1), and several governmental and non‐governmental organizations have put in place initiatives to promote the development and adoption of alternative, non‐animal‐based methods. Meanwhile, scientists highlight that animal research remains indispensable for biomedical progress and that, currently, the value and applicability of many non‐animal methods remains limited (Genzel *et al*, 2020; Homberg *et al*, 2021).

In the United Kingdom, the Wellcome Sanger Institute announced to close its animal facility as a strategic move towards the use of alternative technologies (Else, 2019), a decision that prompted questions within the scientific community. In the Netherlands, neuroscientists and policy makers disagree on the feasibility of the country’s ambitious goal to reduce the number of animal experiments altogether (KNAW, 2019a). In Germany, a prominent neuroscientist has relocated his research activities to China, citing insufficient support in a legal dispute about alleged animal research malpractices (Vogel, 2020). These examples highlight the increased tensions emerging from this important societal debate—which has culminated in a European Union (EU) parliamentary vote in September 2021, in favor of phasing out the use of animals in research, regulatory testing, and education (European Parliament, 2021). Eighteen months earlier, in February 2020, the European Commission released for the first time EU‐wide statistics on animal research, citing improved transparency as a key objective of the new EU directive on animal research (Directive 2010/63/EU). However valuable, such statistics cannot provide the full picture of the status of a research field and the role that animal models play to date. Next to monitoring the use of animals in research, it is equally important to evaluate to what extent the use of non‐animal approaches is able to replace animal research.

We conducted an extensive literature screen to map innovative non‐animal methods in neurodegenerative research. Based on our results, we argue that (i) the vast majority of research into Alzheimer’s and Parkinson’s relies on animal (‐based) models, (ii) animal‐free methods are not necessarily innovative or more human‐relevant, and (iii) there is an urgent need for a more nuanced approach to monitor animal use, set targets, and inform policy discussions.

**Box 1. Animal research policies**

**Across the globe**

The legal framework for animal research has a different historical context in different parts of the world (Institute of Medicine and National Research Council, 2012). In the European Union, the first harmonized legislation on the use of animals for scientific purposes applying to all of its member states dates from 1986. In 2010, a new Directive 2010/63/EU updated and replaced the original legislation to improve animal welfare and anchor the principle of the three Rs, to Replace, Reduce, and Refine the use of animals. The legislation entails rules on species use, procedures, care, housing, and, where applicable, rehoming, as well as authorization requirements and procedures. Directive 2010/63/EU took full effect on 1 January 2013 and is currently under review. Member States can impose stricter measures as long as they do not hinder EU‐wide scientific cooperation and trade.

In the United States, the Animal Welfare Act regulates the treatment of animals in research, exhibition, transport, and by dealers at the federal level. It was approved in 1966 and has been amended several times, most recently in 2013. The Animal Welfare act contains provisions to ensure that animal species used in research receive a certain standard of care and treatment. However, many species are excluded from this legislation (see below “legal definition of a research animal”). The Public Health Service Policy on Humane Care and Use of Laboratory Animals applies to any research facility that receives Public Health Service funds. The National Institutes of Health (NIH) Office of Laboratory Animal Welfare oversees all animal studies funded by the Public Health Service (Institute of Medicine and National Research Council, 2012).

In 2006, China issued the Guidelines on the Humane Treatment of Laboratory Animals. These guidelines officially address animal welfare and protection, with rules on procurement, husbandry, environmental conditions, experimental usage, and transportation. In Japan, the Act on Humane Treatment and Management of Animals promotes animal welfare practices compatible with scientific needs without strictly stipulating them by law. Rather, Japan relies on self‐regulation within each animal facility with administrative guidance and voluntary guidelines to encourage flexible animal research (Ogden *et al*, 2016).

**Legal definition of a research animal**

Animal research legislation is restricted to specific species. In Europe, it only applies to vertebrates. Insects such as *Drosophila melanogaster* or nematodes such as *Caenorhabditis elegans* do not fall under animal research legislation. The non‐vertebrate Octopoda are an exception to this rule and have recently been included within the animal research legislation. Research involving human subjects falls under a different set of regulations, while experiments with other hominids or great apes are strictly forbidden.

The US Animal Welfare Act currently only applies to warm‐blooded animals, but excludes mice, rats, and birds, which along with fish make up 95% of the animals used in research. Other laws, policies, and guidelines (including the Public Health Service Policy on Humane Care and Use of Laboratory Animals) cover additional species or include more specifications for animal care and use, but all refer to the Animal Welfare Act as the minimum acceptable standard.

In China, laboratory animals are defined as any animal bred and reared for experiments or for other scientific purposes. While this definition does not specifically exclude invertebrates, the focus of enforcement is mainly vertebrate animals. The Japanese regulations cover vertebrates, excluding amphibians and fish (Ogden *et al*, 2016).

## Animals in neurodegeneration research

In the European Union alone, almost 10 million animals are used in biomedical experiments each year (European Commission, 2020). In the United States, this number is estimated at 11–23 million animals. (The most recent US statistics fitting the study time frame (2018) list 780,070 lab animals, but mice, rats, and fish are not included. Based on the species distribution for animal research internationally, the total number of vertebrates used in research in the United States is estimated between 11 and 23 million animals (Speaking of Research US Statistics, 2019). To the best of our knowledge, there are no published statistics available to confirm this.) A relatively large proportion are used in neuroscience: in the European Union, nearly 1 million animals are used in basic neuroscience (the highest number for all fields of basic research), and another 300,000 in applied neuroscientific research (second only to applied cancer research; European Commission, 2020). Looking at the scale and scope of neurodegenerative research, Alzheimer and Parkinson research together account for two thirds of all research investments in the field of neurodegeneration (JPND, 2018). We therefore focused our analysis on these two diseases.

**Figure 1:**
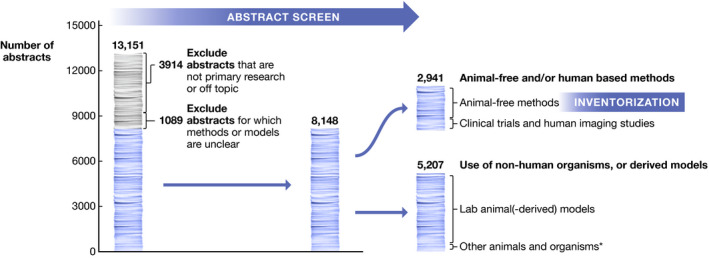
Abstract screen In total, 13,151 abstracts were screened. Abstracts describing non‐primary literature, or research without a direct link to neurodegenerative disease were excluded. The remaining abstracts (6,096 on AD, 3,141 on PD) were further classified based on methodology, using either animal‐based models (*n* = 5,207), human‐based or animal‐free methods (*n* = 2,941), or if insufficient details were provided on the origin of the used model (*n* = 1,089). * “Other animals & organisms” combines animal species to which EU animal research legislation does not apply, as well as other types of organisms such as bacteria and yeast.

In an assignment from the European Commission’s Joint Research Centre, mapping the development and potential value of non‐animal research methods in Alzheimer’s disease (AD) and Parkinson’s disease (PD), we screened the Web of Science from 2013 to 2018 for studies using innovative, animal‐free methods to model or study biological endpoints related to one or both diseases (see Table EV1 for search phrases). Among the 8,148 abstracts that provided information on the experimental methods used, nearly two‐thirds (5,207 abstracts, or 64%) involved models that are, or are derived from, (non‐human) model organisms (Figure 1). Only a small proportion of these (65 abstracts) were restricted to non‐animal model organisms, that is, bacterial or yeast models.

## *In vivo* and *in vitro* animal models

**Figure 2:**
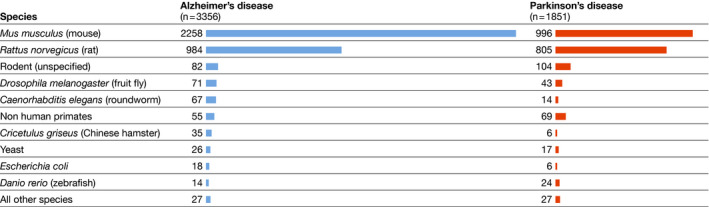
Number of studies relying on animal‐based models, by species The number of studies out of a total of 5,207 abstracts is listed. Some studies involved methods based on different species.

Perhaps unsurprisingly, the majority of animal‐based models relied on rodents. Experiments with mice or mouse‐derived cell lines were identified in 62.5% of all studies using animal‐based or animal‐derived models; 67.3% in the case of AD and 53.8% for PD. Rat‐based models appeared in 34.4% of all abstracts describing animal‐based or animal‐derived models (29.3% for AD, 43.5% for PD). Some abstracts merely specified the use of “rodent” models, which in practice refers to either mouse or rat studies. All other species, including non‐mammalian models such as zebrafish, mammalian species such as pigs and primates, or model organisms that do not fall within the legal definitions of animal research, including *Drosophila* and *C*. *elegans* (see Box 1), represented <5% of the evaluated abstracts in our search (Fig 2).

**Figure 3:**
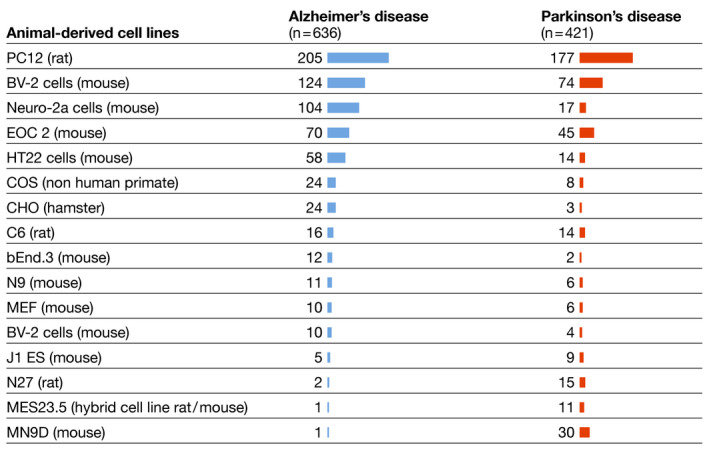
Frequently used cell lines Number of abstracts specifying the use of animal‐derived cell lines. Cell lines that were mentioned in fewer than 10 abstracts in both searches combined are not listed. Some studies involved multiple cell lines.

The actual involvement of living animals in experiments varied significantly and was not always clear from the abstract. For example, 20.3% (*n* = 1,057) of the abstracts involving animal‐derived models mention (immortalized) cell lines of animal origin (636 abstracts for AD, 19%; 421 abstracts for PD, 22.8%; Fig 3). While they originate from animal tissue, they do not imply additional suffering for animals as these cell lines are maintained *in vitro* over many decades and are in many cases purchased from commercial providers. However, in other cellular experiments, researchers derived fresh cells from animal tissue: 936 abstracts (18%) mentioned the use of primary neuronal cultures or brain slices, while 2,505 abstracts (48.1%) referred to animals, embryos or *in vivo* experimental systems, including grafts, transplants, or behavioral analysis.

## The role of non‐animal methods

To map the development and adoption of animal‐free methods, the nearly 3,000 abstracts describing non‐animal methods and models were analyzed in more detail: 671 studies (23%) were human‐based by default (case studies or clinical trials, imaging studies or genetic screens). We classified the remaining abstracts according to model system, that is, (i) biochemical or cell‐free assays, (ii) human‐derived cell lines, (iii) computational or *in silico* models, (iv) human *ex vivo* tissue (brain tissue, cerebrospinal fluid (CSF), blood, saliva, etc.), (v) human primary or stem cells, (vi) microfluidic systems, (vii) 2D and 3D co‐cultures, and (viii) organoids.

For AD, biochemical/cell‐free models represented the most frequently used group of methods (39%), followed by human‐derived cell lines (26%) and computational models (23%). Models based on human‐derived immortalized cells represented the highest fraction (42%) for PD, followed by four types of approaches with approx. equal importance: that is, human primary cells or stem cells (20%), human *ex vivo* tissue and body fluids (19%), biochemical assays (13%), and computational or *in silico* methods (12%).

Both for AD and PD, biochemical or cell‐free assays focused mostly on aspects of protein aggregation, a general feature of neurodegenerative disease. Two of the hallmarks of AD, plaques, and neurofibrillary tangles, are the result of protein aggregation of amyloid‐beta and tau, respectively. In the case of PD, alpha‐synuclein misfolds and aggregates into Lewy bodies. While protein aggregation is an important area of focus for both research fields, there are markedly more developmental efforts and applications in this area for AD‐related research. Human‐derived cell lines, such as SH‐SY5Y cells, are overall the most prominently represented models in the literature we screened. The SH‐SY5Y neuroblastoma cell line has catecholaminergic and dopaminergic neuronal properties and is therefore popular in PD research, as human dopaminergic neurons, the cells mainly affected in PD, are difficult to obtain and maintain as primary cells (Xicoy *et al*, 2017). Experiments performed in these simplified cell systems are considered as indicative. The properties of these cell lines are very different from those of their *in vivo* counterparts: they are transformed and they fail to integrate in complex circuitry or interact with the multicellular environment that typifies the brain (Slanzi *et al*, 2020).

Because of the simplicity of both cell‐free methods and human‐derived cell lines, these models are not likely to ever replace the repertoire and systems complexity of animal‐based methods. They have been used for many decades to answer cell autonomous questions in the absence of tissue, organ, and organismal interactions, but are considered supplementary or are used for preliminary studies.

Similar to biochemical models, computational and *in silico* methods were overwhelmingly focused on protein aggregation, and the same was true for studies using *ex vivo* human tissue. Of the latter, the majority of the studies involved post‐mortem brain tissue, rather than cerebrospinal fluid (CSF) or blood, which means replacement potential for studies using animal models is likely to be restricted.

Human primary and stem cell models are still under full development, with a lot of studies focused on induced pluripotent stem cells (iPSCs) and applications on a broad range of biological endpoints. While in our screen, the majority of primary or stem cell models in AD are used for mechanistic studies, in PD there is a much larger focus on treatment and therapy, including dopaminergic cell replacement.

**Figure 4:**
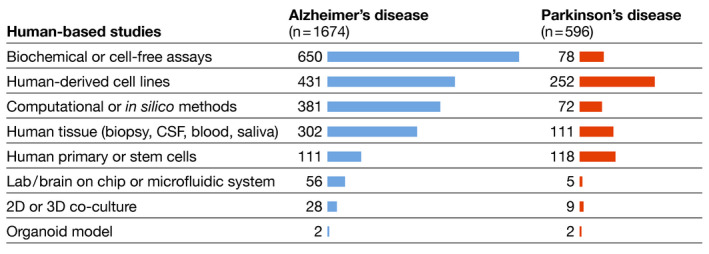
Model systems applied in human‐based and animal‐free studies Some studies applied multiple types of models.

Organoids, 3D‐cultures, or microfluidic systems are three more biologically complex model systems, often highlighted as promising replacements for animal research. However, they were retrieved at much lower frequency in the literature we screened (Fig 4). Many of the AD‐focused brain‐on‐chip or microfluidic models were designed for diagnostic purposes, while for PD the study of protein aggregation was again the most frequently recurring aim. The high throughput that these systems can potentially achieve could be especially valuable in the framework of replacing or supplementing animal experiments. Nevertheless, microfluidic devices are simplified model systems, hence there is the need for physiological, multifunctional, and architecturally complex systems that can better represent the still incompletely elucidated micro‐environment of the central nervous system (CNS) or brain tissue. Co‐cultures, 3D‐cultures, and organoid models are relevant in this context; however, the methods we encountered in our screen were limited and of exploratory nature. An area currently under development relates to organoid vascularization and models for blood‐brain barrier function. These models are promising approaches to reduce animal use, but improved characterization, standardization, and validation against real brain tissue are needed before they can replace animal models for advanced mechanistic studies of disease. Moreover, the better these organoid models become, the more they are going to raise new ethical and regulatory questions (Hyun *et al*, 2020).

## Discussion

Our screen of more than 13,000 abstracts on AD and PD highlights the great reliance on animal research in these fields. Out of the relevant papers that provided sufficient methodological information in the abstract, about two‐out‐of‐three involved (non‐human) animal‐based methods and models, including animal‐based cell lines. We obtained this result despite conducting literature searches that were specifically directed at identifying alternative methods (see Table EV1 and below), suggesting that these findings are an underestimation of the real contribution of animal‐based methods and models in this area.

In terms of animal‐free methods, we have identified areas of focus and interest in relation to methodological development for basic and translational research, some of which are more specific for AD rather than PD or vice versa. One clear example is the prominence of cellular models developed in light of therapeutic applications for PD. Or alternatively, for AD, the much larger focus on development of biochemical methods applied to study protein aggregation. At the same time, we found that promising areas of methodological development covering enhanced biological complexity representing CNS were surprisingly underdeveloped. Almost three in four animal‐free studies used non‐innovative methods or models, such as basic biochemistry and immortalized cell lines of human origin.

Our literature screen has several limitations. First, we did not include the entire body of literature on Alzheimer’s and Parkinson’s disease. In line with the scope of the JRC mandate (Witters *et al*, 2021), our search strategy was targeted toward animal‐free methods and a selection of disease features, resulting most likely in an enrichment of such methods (Table EV1). By basing ourselves on scientific literature, our analysis also suffers from publication bias, as certain methods may be under‐ or overrepresented in experiments that are eventually published versus those that are not. Second, the first phases of our literature review were limited to abstract screening. As is clear from Fig 1, a large proportion of studies had insufficient methodological information listed in the abstract to conclude on the type of methods and models used. However, also the full text screening regularly lacked essential information, for example, relating to cellular sources or animal species. Third, to reflect a recent status of the field, we analyzed literature in a 5‐year window between 2013 and 2018. This window limits our options to discern trends over time.

While there is a huge practical and ethical difference between an *in vivo* experiment and the use of an immortalized cell line of animal origin, drawing the line between *animal* and *animal‐based* models proved difficult. This was not only due to the often‐limited methodological information provided in publications, but also because the distinction is not always clear‐cut. We believe that combining the current exercise to map *model* use is of complementary value to the more routinely collected statistics of *animal* use.

**Box 2. Animal research in biomedical sciences**

Animal research extends far beyond the fields of AD and PD, and in addition to *in vitro* studies, it is an important aspect of pre‐clinical research. Next to neuroscientific research, cancer research and immunological research account for the largest proportion of animal use in the European Union (European Commission, 2020).

**COVID‐19 vaccines and treatments**

In response to the global COVID‐19 pandemic, the World Health Organization assembled an international panel to develop animal models to accelerate the testing of vaccines and therapeutic agents. Our expanding knowledge of SARS‐CoV‐2, the development of vaccines and of treatments all relied heavily on animal models, including, mice, hamsters, ferrets, and non‐human primates (Muñoz‐Fontela *et al*, 2020).

**Immunotherapy to treat cancer**

Employing the body’s own immune cells to target malignant tumors is the basic premise of immunotherapy. This approach has saved the lives of countless cancer patients and has led to the Nobel prize for two of its pioneers in 2018. From the basic research on T‐cell biology through seminal proof‐of‐concept pre‐clinical work as a cancer treatment, mice have played an instrumental role (Waldman *et al*, 2020).

**Deep brain stimulation for Parkinson’s disease**

Non‐human primates were essential in the development of deep brain stimulation treatments for Parkinson’s disease as well as brain‐controlled prosthetic devices for lost limbs. Deep brain stimulation involves the application of high‐frequency stimulation, in the case of Parkinson’s disease of the subthalamic nucleus, considerably reducing motor symptoms in patients (Benazzouz *et al*, 2016).

## Future perspectives on the reduction of animal research

### Are we making progress?

Governmental reports tell us the absolute numbers of animals used in research—in a given country or year, or for a given purpose. Whether or not the neurodegeneration field in general is moving towards a decrease in animal testing and increase in alternative, human‐based methods is difficult to assess without considering a temporal dimension. Because of legislative differences, it is already difficult to compare animal use statistics across the globe (Box 1), let alone to gauge the relative use of animal versus non‐animal‐based methods, and even more complex to compare this between fields or over time. We did not identify significant changes in relative distribution over the 5‐year period; however, our findings show that even if such a trend would exist, we are still a long way from partial, let alone complete replacement, of animal research when it comes to neurodegeneration research. Abruptly stopping the use of animals would significantly cripple these research fields (in the EU).

Furthermore, the development of more human‐centered methods and models, which would arguably have a higher translational value, does not correspond directly with a move from animal to animal‐free methods. For example, animal models themselves are increasingly humanized to combine the best of both worlds (Aartsma‐Rus & Van Putten, 2020). Such animal models carry functioning human genes, cells, tissues, or even organs. Examples in AD and PD include xenotransplantation of human stem cells in mouse brain (Espuny‐Camacho *et al*, 2017; Hoban *et al*, 2020). Additionally, relative and absolute numbers may tell a different story when a research field is expanding, as may very well be the case for neurodegeneration—a field suffering from historic underinvestment and increasing societal burden (Nichols *et al*, 2019; Aerts *et al*, 2021). Thus, even if the number of animals per researcher will decrease, the absolute numbers are likely to increase for the years to come.

### When reducing animal research, which aims are realistic?

The massive reliance on animal methods suggests that a move towards animal‐free research—without shifting current focus or goals—is currently unrealistic. The disease focus or biological endpoint of the innovative inventoried non‐animal‐based methods and models screened in this study is largely on protein (dys)function (51.6%). None of these models assess cognitive, locomotor, or other types of higher‐order brain function, which are the most relevant clinical features when studying AD and PD (or most other neuronal diseases). iPSCs and iPSC‐based 3D‐cultures or brain‐on‐chip approaches offer the advantage of human and patient‐specific modeling. They can replace aspects of animal‐based *in vitro* and *in vivo* experiments on neurological disease, for example, for disease mechanisms that manifest in a specific cell type or for the identification of new candidate drug targets. However, these models are limited in terms of complexity and thus inadequate for read‐outs of system‐level brain activity or behavior. In AD and PD research, examples include the study of learning, memory, social interaction, sleep, locomotor, or executive function. This is not likely to change soon, given the incomplete knowledge of these complex functions of the brain, and thus the difficulty of modeling these *in vitro*.

This observation is in line with the conclusions of the Royal Dutch Academy, who reported that “in addition to experimental methods that require no or fewer animals, animal experimentation is and will continue to be an indispensable and important basic component of that mix in the foreseeable future, and necessary for doing high‐quality fundamental brain research” (KNAW, 2019b). In addition to further standardization and maturation of organoid models and the use of computational models, which offer growing, but inevitably limited opportunities for animal experiment replacement, the Royal Dutch Academy points to invasive and non‐invasive research in humans, including the repurposing of human tissue, to replace animal research to some extent.

In light of these conclusions, bold statements on animal replacement should be accompanied by a realistic assessment of the effects on research, especially in the case of neuroscience. A forced reduction in animal experiments (by legal means) to stimulate development of alternative methods also comes with a risk, if the promise of improved humanized models cannot be realized. Policymakers setting the research agenda should carefully consider the compatibility of ambitions to reduce animal experiments and to find a cure, for example, for AD or PD in the next decade (see Box 2 for examples of animal research enabling medical breakthroughs).

Important in this regard is that while policy is local, research is global. Pursuing accelerated animal replacement through stricter regulations locally, may inadvertently encourage so‐called ethics dumping, that is, exporting unethical and/or unlawful practices to other settings. With animal research policies differing across the globe (Box 1), would replacement of animal research in practice simply mean displacement of animal research? Furthermore, it remains unclear how regions with stricter animal research regulations will adopt results or therapies generated elsewhere, with animal experiments.

### How should we monitor progress?

It is difficult to cover an entire field and obtain accurate numbers on method development, let alone use, on such broad terms as animal or non‐animal use. As exemplified above, immortalized animal cell lines cannot be compared to *in vivo* experiments, but at the same time cell‐free biochemical *in vitro* studies often also still rely on animal‐derived antibodies, organoids may be derived from animal primary cells, etc. Thus, animal‐based and animal free approaches are two ends of a spectrum, not a clear‐cut dichotomy.

If further advancement of the 3Rs in neurodegeneration research is to be successful, we need much better monitoring of methods, not only statistics on animal use, to guide realistic and reliable policies. This monitoring should not come at the expense of researchers, who—in Europe and many other parts of the world—currently already face an increasing bureaucratic burden (and thus cost) when using animals to advance disease research and therapy development. It goes without saying that clear ethical guidelines and a legal framework are indispensable and that researchers are rightly held accountable when designing and conducting experiments. However, we advocate treating animal researchers as partners—not opponents—in the search for and development of better models for disease.

Several initiatives have been launched both locally and internationally, to collect and implement animal‐free methods—including the inventory we developed through our literature search (Witters *et al*, 2021). However, the merits of such databases relate primarily to potential gains in dissemination and require to be placed in perspective with animal‐based methods as well, to gain a more complete understanding of the status of the field—as we aimed to do here.

In conclusion, when investing in the development of animal‐free methods and promoting the use, validation and acceptance of alternative approaches, better monitoring of their applications and value relative to existing or newly developed animal methods is essential. In which type of experiment or setting can such new models be most valuable or predictive for brain (disease) research? Forced overstimulation of the development of non‐animal methods could lead to the promotion of low‐complexity research models, with little added value for disease understanding.

## Materials and Methods

We retrieved abstracts from peer‐reviewed publications dating between 2013 and 2018, that combined a specific mention of (i) either AD or PD, (ii) a relevant biological endpoint to this disease, and (iii) a method or model system (e.g. “cell model,” “comput*”) from Web of Science. Non‐English and/or non‐primary literature (e.g., reviews) was excluded. Search phrases are listed in Table EV1. All abstracts were randomly distributed among four assessors and evaluated for scope, fit and methodology (interrater reliability: Fleiss kappa of 0.46), and further categorized based on the use of animal‐based versus animal‐free or human‐based models. Animal‐based abstracts were screened for species and/or cell type using automated text‐mining (manual quality control with interrater reliability: Cohen’s kappa of 0.97). The abstracts on animal‐free or human‐based models were further prioritized according to (i) the type of study (i.e., methodological development or validation versus application of routine or existing methods), (ii) the type of model (*ex vivo* material, cell culture, biochemical, etc.), and (iii) if applicable, the cell type and/or biological endpoint or read‐out. For all papers categorized with high priority (i.e., publications containing sufficiently novel alternative methods) full texts were screened and, where appropriate, methodological details were collected. The resulting inventory gathered information pertaining to the “what,” “why,” and “how” of a given method, alongside qualitative information on its relevance, scope, and potential and has been published as a report deliverable for JRC project JRC124723.

## Supporting information



Table EV1
