# Supplementary material for: Do we still need animals? Surveying the role of animal‐free models in Alzheimer’s and Parkinson’s disease research
Source: EMBO J. 2022 Feb 24;41(6):e110002. doi: 10.15252/embj.2021110002 (PMC8922267; doi:10.15252/embj.2021110002)
Supplement: Supplementary file 1 — Table EV1 [file EMBJ-41-e110002-s001.docx]

**Table EV1: Literature search phrases for Alzheimer’s disease and Parkinson’s disease publications**

| Set | **Run Search: "Alzheimer_20180904": 8411 hits** |
| --- | --- |
|  | Custom year range 2013-2018; Language: English; Sources: Science Citation Index Expanded (SCI-EXPANDED) & Emerging Sources Citation Index (ESCI); Type of document: article, review |
| #4 | #3 AND #2 AND #1 |
|  | DocType=All document types; Language=All languages; |
| #3 | (TS= ("in vitro" OR "ex vivo" OR "cell model" OR "cell culture" OR "stem cell" OR "cell line" OR "primary cell*" OR "brain tissue*" OR "brain slice*" OR "brain sample*" OR "cerobrospinal fluid*" OR *chip* OR microfluid* OR microdevice* OR organoid* OR *spheroid* OR "comput* model*" OR "comput* simulat*" OR "comput* method*" OR "comput* tool*" OR "model* approach*" OR "in silico" OR QSAR OR "molecular field analy*" OR "molecul* dock*" OR bioinformat* OR "data acquisit*" OR "data mining" OR "enrichment analy*" OR "differential analy*" OR "predict* model")) AND LANGUAGE: (English) AND DOCUMENT TYPES: (Article OR Review) |
|  | DocType=All document types; Language=All languages; |
| #2 | (TS= ("amyloid*” OR “Aβ” OR "tau" OR "total tau" OR "phosphor-tau" OR “tau phosphoryl*” OR "neurofibrillary tangle" OR "NFT" OR "oxidat* stress" OR "neuroinflammat*" OR "microglial dysfunction" OR "excitotoxicit*" OR "lysosom* defect" OR "mitochondrial defects” OR “mitochondrial dysfunction" OR "axon* transport*")) AND LANGUAGE: (English) AND DOCUMENT TYPES: (Article OR Review) |
|  | DocType=All document types; Language=All languages; |
| #1 | (TS=Alzheimer*) AND LANGUAGE: (English) AND DOCUMENT TYPES: (Article OR Review) |
|  | DocType=All document types; Language=All languages; |

| Set | **Run Search: "Parkinson_20180904": 4740 hits** |
| --- | --- |
|  | Custom year range 2013-2018; Language: English; Sources: Science Citation Index Expanded (SCI-EXPANDED) & Emerging Sources Citation Index (ESCI); Type of document: article, review |
| #4 | #3 AND #2 AND #1 |
|  | DocType=All document types; Language=All languages; |
| #3 | (TS= ("in vitro" OR "ex vivo" OR "cell model" OR "cell culture" OR "stem cell" OR "cell line" OR "primary cell*" OR "brain tissue*" OR "brain slice*" OR "brain sample*" OR "cerobrospinal fluid*" OR *chip* OR microfluid* OR microdevice* OR organoid* OR *spheroid* OR "comput* model*" OR "comput* simulat*" OR "comput* method*" OR "comput* tool*" OR "model* approach*" OR "in silico" OR QSAR OR "molecular field analy*" OR "molecul* dock*" OR bioinformat* OR "data acquisit*" OR "data mining" OR "enrichment analy*" OR "differential analy*" OR "predict* model")) AND LANGUAGE: (English) AND DOCUMENT TYPES: (Article OR Review) |
|  | DocType=All document types; Language=All languages; |
| #2 | (TS= ("α synuclein" OR "alpha synuclein" "Lewy bod*" OR “dopamine*” OR "substantia nigra" OR striatum OR "synap* dysfunction*" OR "synap* defect*" OR "protein aggreg*" OR "protein misfold*" OR "MPP+" OR "neuron cell death" OR "oxidat* stress" OR "neuroinflammat*" OR "microglial dysfunction" OR apopto* OR "excitotoxicit*" OR "autophag*" OR "lysosom* defect" OR "mitochondr* dysfunction*" OR "axon* transport*")) AND LANGUAGE: (English) AND DOCUMENT TYPES: (Article OR Review) |
|  | DocType=All document types; Language=All languages; |
| #1 | (TS=Parkinson*) AND LANGUAGE: (English) AND DOCUMENT TYPES: (Article OR Review) |
|  | DocType=All document types; Language=All languages; |
